# The Poor Man’s Gems

**Published:** 1892-06

**Authors:** 


### THE POOR MAN'S GEMS.

H. B. S.

Oh, ye whose hands are brown from toil,
  Give heed to what I say;
Though ye must bend with care awhile,
  Your hearts may well be gay,
You cannot boast of wealth or power;
  Yet God is just and kind;
You own a great, a mighty dower,
  A free and noble mind.

When Envy would your thoughts engage
  Look in Contentment's store;
Sail on; we've men in this great age
  Now rich who once were poor.
Why will you fret and waste your time?
  Up, man! to play your part;
You own a gem unknown to crime—
  A true and honest heart.

Then ne'er repine, my fellow men;
  I work as well as you;
Why should the forge despise the pen?
  Both have one end in view.
The poor should link both heart and soul,
  Like brothers on the sod,
If they would reach sweet honor's goal
  And own one master—God.

